# Simultaneous Detection of Five Pathogens from Cerebrospinal Fluid Specimens Using Luminex Technology

**DOI:** 10.3390/ijerph13020193

**Published:** 2016-02-04

**Authors:** Linfu Zhou, Rui Wu, Xiaodan Shi, Dongyun Feng, Guodong Feng, Yining Yang, Wen Dai, Ting Bian, Tingting Liu, Ying He, Ming Shi, Gang Zhao

**Affiliations:** 1 Department of Neurology, Xijing Hospital, Fourth Military Medical University, Xi’an 710032, China; zhoelfu@163.com (L.Z.); wurui1124@126.com (R.W.); shixd999@163.com (X.S.); fengdy@fmmu.edu.cn (D.F.); fgd2000@fmmu.edu.cn (G.F.); yiningyang80@163.com (Y.Y.); wwdd126@126.com (W.D.); bianting-44@163.com (T.B.); miracle6tt@163.com (T.L.); skyblue20090802@163.com (Y.H.); 2 Department of Neurology, Third Hospital of People’s Liberation Army, Baoji 721004, China

**Keywords:** Luminex technology, multiplex PCR, central nervous system infections, cerebrospinal fluid

## Abstract

Early diagnosis and treatment are crucial for the outcome of central nervous system (CNS) infections. In this study, we developed a multiplex PCR-Luminex assay for the simultaneous detection of five major pathogens, including *Mycobacterium tuberculosis*, *Cryptococcus neoformans*, *Streptococcus pneumoniae*, and herpes simplex virus types 1 and 2, which frequently cause CNS infections. Through the hybridization reaction between multiplex PCR-amplified targets and oligonucleotide “anti-TAG” sequences, we found that the PCR-Luminex assay could detect as low as 10^1^–10^2^ copies of synthetic pathogen DNAs. Furthermore, 163 cerebrospinal fluid (CSF) specimens from patients with suspected CNS infections were used to evaluate the efficiency of this multiplex PCR-Luminex method. Compared with Ziehl-Neelsen stain, this assay showed a high diagnostic accuracy for tuberculosis meningitis (sensitivity, 90.7% and specificity, 99.1%). For cryptococcal meningitis, the sensitivity and specificity were 92% and 97.1%, respectively, compared with the May Grunwald Giemsa (MGG) stain. For herpes simplex virus types 1 and 2 encephalitis, the sensitivities were 80.8% and 100%, and the specificities were 94.2% and 99%, respectively, compared with Enzyme Linked Immunosorbent Assay (ELISA) assays. Taken together, this multiplex PCR-Luminex assay showed potential efficiency for the simultaneous detection of five pathogens and may be a promising supplement to conventional methods for diagnosing CNS infections.

## 1. Introduction

CNS infections caused by viruses, bacteria, fungi, parasites and prions still remain diseases with significant morbidity and mortality [1,2]. In many developing countries affected by endemic diseases such as tuberculosis, HIV/AIDS, and cryptococcal meningitis, delayed diagnosis, treatment and high economic burden often lead to avoidable deaths or severe sequelae [3]. To reduce the mortality and improve the cure rates, it is vital to screen and identify pathogens in clinical specimens from patients as early as possible [4,5]. Early and accurate diagnosis will support physicians with the selection of the appropriate antimicrobial agents [6,7]. Most laboratory methods for diagnosing CNS infections are mainly based on the identification of pathogens by staining or cultures, the detection of specific antigens and antibodies, or the examination of pathogen nucleic acids with polymerase chain reaction (PCR) in CSF [8,9]. Microscopy of CSF is a widely applied rapid diagnostic technique, however, the time investment is not feasible in most busy routine diagnostic laboratories, where it has been shown increasing the examination time of CSF smears is necessary for improving sensitivity [10,11,12]. Although diagnosis based on culture is the reference standard, the clinical value of culture technique is limited due to its low sensitivity and long time consumption [13]. Previous studies have showed that multiplex PCR shows high sensitivity and specificity for the detection of pathogens [14,15,16], however, this method may be difficult to implement with rigorous quality controls, and becomes more challenging notably in terms of low numbers of microorganisms in CSF specimens [13,17]. Moreover, the throughput of PCR method is relatively limited since only a few genes can be detected in a single reaction [14,16,17,18,19]. 

Luminex technology is a multi-plex assay characterized with high-throughput, accuracy, high speed, sensitivity and specificity, which can qualitatively and quantitatively analyze and report up to 100 different reactions in a single reaction vessel [20,21,22,23,24]. Previous studies have reported that this technology offered a new platform for high-throughput nucleic acid detection which is being used in infectious diseases [25,26,27,28].

In the present study, we developed a rapid, high-throughput diagnostic method integrated multiplex PCR and Luminex technology for detecting five pathogens, including *M. tuberculosis*, *C. neoformans*, *S. pneumoniae*, herpes simplex virus types 1 (HSV-1) and 2 (HSV-2), which account for more than 70% of CNS infections in Northwest China. Moreover, we applied this method to detect the five pathogens simultaneously in a large number of CSF specimens from suspected CNS infection patients in Xijing Hospital, one of the biggest hospitals in China’s Northwest Region, where approximately 120,000 outpatients with neurological diseases are treated annually, among which around 4000 were suspected of CNS infections.

## 2. Materials and Methods 

### 2.1. Viral and Bacterial Strains

Five pathogenic strains, including *M. tuberculosis* strain H37Rv, *C. neoformans* strain H99, *S. pneumoniae* strain D39 (Center for Clinical Laboratory Medicine of Xijing Hospital, the Fourth Military Medical University), HSV-1 strain SM44 (Department of Microbiology, the Fourth Military Medical University), HSV-2 strain MS (The National Center for Microbial Engineering Detection, Northwest University), were used in the study.

### 2.2. Clinical Specimens

A total of 163 CSF specimens were collected from 1996 to 2013 at the CSF Laboratory of Xijing Hospital for Central Nervous Infectious Diseases in Shaanxi Province, China. Briefly, 2 mL of CSF was centrifuged at 3000× *g* for 15 min, and the sediment was smeared on slides as previously described [29]. All smears were stained by the Ziehl-Neelsen stain [29,30], MGG stain [31] and Gram stain for the presence of *M. tuberculosis*, *C. neoformans* and *S. pneumoniae* and observed under a light microscope. Furthermore, all the CSF specimens were also tested for HSV-1 and HSV-2 with a commercial ELISA assay. Among them, 54 CSF specimens were obtained from patients with definite tuberculosis meningitis [32], defined as a clinical syndrome consistent with tuberculosis meningitis, with Ziehl-Neelsen stain positive or *M. tuberculosis* isolated in CSF Mycobacteria Growth Indicator Tube (MGIT 960) system culture. Twenty five CSF specimens from patients with cryptococcal meningitis were definitely diagnosed by MGG stain. Twenty seven CSF specimens from patients with HSV-1 and HSV-2 encephalitis were identified by a commercial ELISA assay. The remaining 57 CSF specimens, showing negative results by either Ziehl-Neelsen stain, MGG stain, Gram stain or ELISA detection, were also used in this study. In addition, 20 CSF specimens from non-infected patients such as cerebral infarction, cerebral hemorrhage, Alzheimer’s and Parkinson’s disease were also analyzed.

### 2.3. Primer 

**Figure A1:**
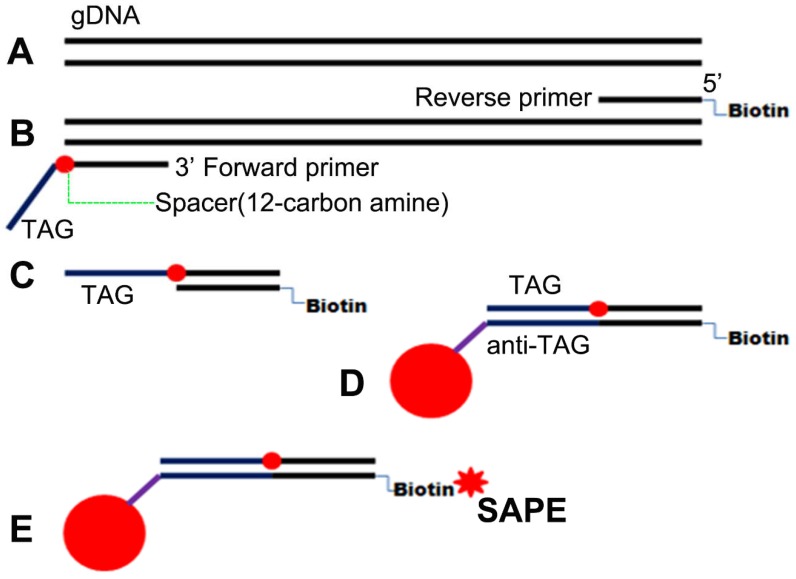
Schematics for multiplex PCR-luminex assay. (**A**) Genomic template DNA is firstly extracted from CSF specimens; (**B**) Reverse primer and forward primer are then labeled with biotin and an unique 24 base oligonucleotide “TAG” sequence at the 5’ terminus, respectively. (C-E) When this “TAG” primer is combined with 5’ biotinylated reverse primer, a sequence specific double-stranded biotinylated amplicon is generated; (**C**) which is captured by a MagPlex-TAG microsphere through the hybridization of its “TAG” to its complementary “anti-TAG” sequence; (**D**) and then the captured amplicon is labeled with streptavidin-R-phycoerythrin (SAPE); (**E**) Finally, the fluorescent signal associated with each microsphere is measured by Luminex 200 analyzer.

Four sets of primers were designed using the Oligo6.5 software. The primer for *S. pneumoniae* was cited in a previous study [33]. The GenBank reference sequence GIs applied for the primer design were CP009480.1 for *M. tuberculosis*, L38588.1 for *C. neoformans*, AF467249.1 for *S. pneumoniae*, X14112.1 for HSV-1, and Z86099.2 for HSV-2. Each forward primer was modified by an unique 24 base oligonucleotide “TAG” sequence at 5’ terminus which is captured to MagPlex-TAG microsphere with a complementary “anti-TAG” sequence. The “TAG” sequence was separated from the sequence specific portion of the primer with an internal spacer for 12-carbon amine. Reverse primer was biotinylated at 5’ terminus (see Appendix Figure A1). The MagPlex-TAG microspheres were purchased from Luminex Corporation. All primers were synthesized by Takara Biotechnology (Dalian, China), and their sequences are listed in Table 1. 

**Table 1:** The specific primers used in multiplex PCR with Luminex assay.

Organism	GenBank Accession No.	Target Gene	Oligonucleotide Sequence of Primer (5’–3’)	Length (bp)	Position (5’–3’)
***M.*** ***tuberculosis***	CP009480.1	IS986	F- CTTAACATTTAACTTCTATAACAC-12C-CGTGAGGGCATCGAGGTGGCR-biotin-GCGTAGGCGTCGGTGACAAA	245	889650–889894
***C*** ***.*** ***neoformans***	L38588.1	URA	F- CACTTAATTCATTCTAAATCTATC-12C-TGTCCTAACCAGTGCGACAGCGATGR-biotin-GTACTTCCTGACCTCTTGCAGCTCC	360	390–749
***S. pneumoniae***	AF467249.1	lytA	F- ATTAAACAACTCTTAACTACACAA-12C-CGCAATCTAGCAGATGAAGCAGGTT R-biotin-AAGGGTCAACGTGGTCTGAGTGGTT	124	328–451
**HSV-1**	X14112.1	Gene 42	F- TACATTCAACACTCTTAAATCAAA-12C-GCCGTTGAGCTAGCCAGCGA R-biotin-GTGCTGGTGCTGGACGACAC	257	93557–93813
**HSV-2**	Z86099.2	TK	F- ACTACTTATTCTCAAACTCTAATA-12C-GTAAGCGCGGGCCAAAGGATR-biotin-TCAAACACGGAAGCCCGAAC	234	46635–46868

F = forward, R = reverse. Unique 24 base oligonucleotide “TAG” sequences and 12-carbon amine containing group are indicated by underline and bold, respectively. R-primers were biotinylated at 5’ terminus. HSV-1 and HSV-2, herpes simplex virus types 1 and 2.

### 2.4. Plasmid Preparation

We synthesized 245, 360, 124, 257 and 234 bp sequences of the conserved regions of *M. tuberculosis, C.* *neoformans, S. pneumoniae*, HSV-1 and HSV-2, respectively. The nucleic acids were cloned into pUC57 plasmid (Sangon, Shanghai, China). Then these plasmids were transformed into *Escherichia coli* (DH5a), and plasmid DNAs were extracted with E.Z.N.A DNA mini Kit I (Omega, Doraville, GA, USA). OD value was measured by UV spectrophotometer (Eppendorf, city, Germany). Copy numbers were calculated from the formula: (6.02 × 10^23^) × (X ng/μL × 10^−9^)/(DNA length × 660) = copies/μL (X was the concentration of the plasmid). For subsequent experiment, five plasmid DNAs used in this study were prepared with 10-fold serial dilutions.

### 2.5. DNA Extraction and PCR Amplification

CSF specimens with volumes ranged from 200 to 400 µL were stored at −20 °C. After thawing the CSF samples, they were centrifuged for 15 min at 13,000 ×*g* and the supernatant was removed. Four hundred microliters of distilled water was added, and the pellet was resuspended. And then, pathogen genomic DNA was extracted by using E.Z.N.A. MicroElute Genomic DNA Kit (Omega, Doraville, GA, USA), according to the manufacturer’s instructions. DNA was eluted in 30 µL elution buffer and was subsequently stored at –20 °C, and thawed once just before use. The multiplex PCR amplification was performed in a total volume of 25 µL containing the following: 1 × multiplex master mix (Qiagen, Ballerup, Denmark), 8 μL genomic DNA template and 200 nM of each primer (Takara, Dalian, Liaoning, China) (Table 1), Positive control just needed 1 μL (80–130 ng) plasmid DNA and distilled water was used as a negative control. Amplification conditions were as follows: 95 °C for 5 min, followed by 35 cycles of 94 °C for 30 s, 59 °C for 90 s, and 72 °C for 30 s, and a final extension step of 72 °C for 10 min, and stored at 4 °C. 

### 2.6. PCR Product Identification and Luminex Assay

PCR products were visualized by 1.5% agarose gel electrophoresis to examine the amplified DNA. The target PCR products for *M. tuberculosis, C.* *neoformans, S. pneumoniae,* HSV-1 and HSV-2 were 245, 360, 124, 257 and 234 bp, respectively. And these target gene fragments could also be obtained by PCR amplification of the genomic DNA from five pathogen strains. 

The PCR-Luminex assay procedure is available at the Luminex Corporation website (http://info.luminexcorp.com/download-the-xmap-cookbook). Briefly, MagPlex-TAG microspheres sets were prepared and resuspended by sonication and vortex for approximately 20 s, and then a Working Microsphere Mixture was prepared by diluting the MagPlex-TAG microspheres stocks to 100 microspheres of each set/µL in 2 × Tm Hybridization Buffer (0.4 M NaCl, 0.2 M Tris, 0.16% Triton X-100, pH 8.0, Molecular Grade dH_2_O) by vortex for 20 s. Subsequently, 25 µL of MagPlex-TAG microspheres Mixture was added in each sample or background well and 25 µL distilled water was added in each background well. For each sample well, 7 µL amplified biotinylated PCR products and distilled water were added to a total volume of 50 µL. The mixtures were incubated at 96 °C for 90 s and then hybridized in a thermocycler for 30 min at 37 °C.

After the beads were pelleted, the supernatant was removed by using vacuum manifold (Luminex, Austin, TX, USA). And the pelleted microspheres were washed twice with 200 μL 1 × Tm Hybridization Buffer. Fresh reporter mix (75 µL) was prepared by diluting Streptavidin, R-Phycoerythrin Conjugate (SA-PE, Invitrogen, Carlsbad, CA, USA) to 5 µg/mL in 1 × Tm Hybridization Buffer. The Bead-PCR product-SAPE mixtures were incubated at 37 °C in dark for 15 min and analyzed on the Luminex 200 analyzer (Luminex Corporation) according to the system manual. The sample size was set to 50 µL with a minimum of 50 beads per target analyzed. Each microsphere was analyzed by a red laser which identified the color of the microsphere and a green laser which analyzed the surface content of the SA-PE bound to the hybridized PCR products [23,24,33]. 

### 2.7. Determination of Cutoff Values and Data Analysis

The data were reported as median fluorescence intensity (MFI) and exported from the Luminex instrument. The determination of cutoff values were based on previous studies [33,34], which obtained from the median fluorescence intensities of 55 no-template controls (NTCs) run during the study period. We calculated a corrected MFI (cMFI), which normalizes to negative control fluorescence as follows: cMFI = (MFI (sample) − MFI (negative control))/MFI (negative control). The cutoff cMFI chosen was three times the 95th percentile for the each pathogen. The MFI values of samples were shown as mean ± SEM and then analyzed using OriginPro 8.0 software. Additionally, the consistency check was performed using Cohen’s kappa test for SPSS17.0 software.

## 3. Results

### 3.1. Specificity of Primers

Using plasmid DNAs, five sets of specific primers were confirmed by single nucleic acid amplification. Single PCR results showed that all the target gene fragments can be obtained by 1.5% agarose gel electrophoresis, and no cross-reactions were found (data not shown).

### 3.2. Determination of Amplification Conditions for Five-Plex PCR

**Figure 1:**
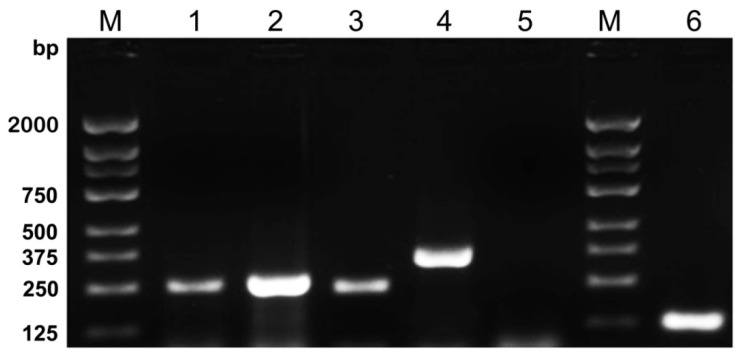
Agarose gel electrophoresis showed the results of five-plex PCR amplification followed by *M. tuberculosis,* HSV-1, HSV-2, *C.* *neoformans* and *S. pneumoniae* plasmid DNAs, respectively. Each plasmid DNAs was successfully amplified by five-plex PCR Reaction System. Lanes are as follows: M, DNA Marker 2000; 1, *M. tuberculosis* 245 bp; 2, HSV-1 257 bp; 3, HSV-2 234 bp; 4, *C.* *neoformans* 360 bp; 5, negative control; 6, *S. pneumoniae* 124 bp.

Next we applied a five-plex PCR Reaction System to amplify the plasmid DNAs to test the efficiency of the system. Agarose gel electrophoresis analysis (Figure 1) showed that this multiplex PCR system was feasible and could accomplish an effective amplification for each plasmid.

### 3.3. Analytical Specificity of PCR-Luminex Assay

**Figure 2:**
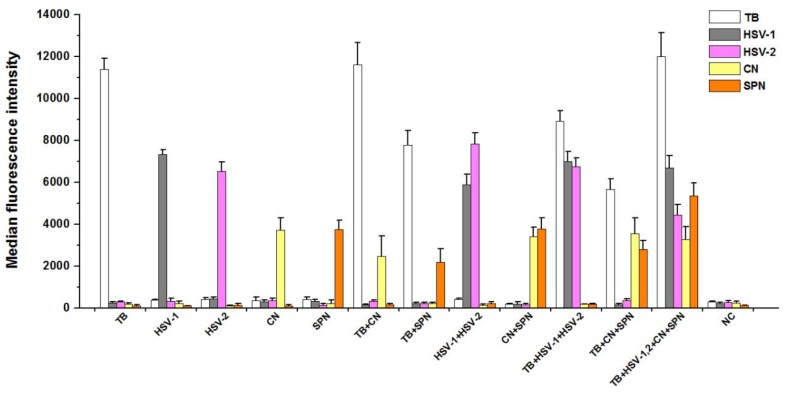
Detection of target products by the five-plex PCR-Luminex showed a high specificitity. Distilled water was used as the negative control (NC). Each bar represents the average MFI of triplicate samples. The error bars indicate standard deviations. TB, *M. tuberculosis*; CN, *C. neoformans*; *SPN*, *S. pneumoniae*; HSV-1 and HSV-2, herpes simplex virus types 1 and 2.

The Luminex assay of multiplex PCR products for each plasmid DNAs (Figure 2) showed a single and specific fluorescence signal. For two plasmid DNAs as templates, the two different specific fluorescence values were detected through multiplex PCR-Luminex assay. Moreover, multiple specific fluorescence values could be obtained from various mixed templates, indicating that PCR products were only hybridized with oligonucleotide “anti-TAG” sequences on the corresponding microspheres in the same testing system. 

### 3.4. Analytical Sensitivity of PCR-Luminex Assay

The sensitivity of the PCR-Luminex assays was assessed by testing serial dilutions of five plasmid DNAs for *M. tuberculosis*, HSV-1, HSV-2, *C.* *neoformans* and *S. pneumoniae*. The tests were performed at each concentration of plasmid, with distilled water used as NC. The results showed that the limit of detection was 10^2^ plasmid copies for *M. tuberculosis*, *C.* *neoformans,* HSV-2 and *S. pneumoniae* and 10^1^ plasmid copies for HSV-1 (Table 2). The sensitivity of the assay for HSV-1 was higher than the others. All detects were evaluated with the single-plex amplicons in multiplex PCR and performed for three times. 

**Table 2:** Analytical sensitivity of serial dilution plasmid.

Organism	Plasmid Quantity (Copies)	cMFI
*M. t* *uberculosis*	10^8^	16.95 ± 0.45
	10^6^	10.06 ± 0.22
	10^4^	7.49 ± 0.30
	10^2^	6.79 ± 0.20
	10^1^	0.93 ± 0.07
HSV-1	10^2^	11.41 ± 0.72
	50	6.56 ± 0.20
	10	5.30 ± 0.31
	5	2.30 ± 0.12
	1	2.45 ± 0.15
HSV-2	10^8^	17.72 ± 0.59
	10^6^	7.14 ± 0.38
	10^4^	6.19 ± 0.14
	10^2^	3.49 ± 0.07
	10^1^	2.25 ± 0.02
*C.* *neoformans*	10^8^	34.80 ± 0.21
	10^6^	31.86 ± 0.18
	10^4^	28.29 ± 0.45
	10^2^	9.62 ± 0.12
	10^1^	1.23 ± 0.10
*S. pneumoniae*	10^8^	8.78 ± 0.47
	10^6^	6.15 ± 0.36
	10^4^	4.94 ± 0.29
	10^2^	3.77 ± 0.22
	10^1^	2.18 ± 0.14

Cutoff cMFI = 3.0. All samples were performed in triplicate. If the results from a duplicate sample analysis exceeded the Cutoff cMFI, the sample was defined as PCR-Luminex assay positive.

### 3.5. Assessment of PCR-Luminex Performance Using CSF Specimens

Then we applied this multiplex PCR-Luminex method to examine 163 CSF specimens from patients with suspected CNS infections. We found that according to the cutoff cMFI, 113 CSF specimens were positive by PCR-Luminex assay. Of these, 50 were positive for *M. tuberculosis*, 27 were positive for *C. neoformans*, 29 were positive for HSV-1 and two were positive for HSV-2. In addition, five specimens infected with *S. pneumoniae* were also found. The detection results for pathogens obtained by microscopy, ELISA and PCR-Luminex assay are shown in Table 3, Table 4 and Table 5. For tuberculosis meningitis, 49 of 54 specimens confirmed by Ziehl-Neelsen stain were detected positive following PCR-Luminex assay which were true positive. Among the CSF specimens showing negative results by either Ziehl-Neelsen stain, MGG stain, Gram stain or ELISA detection, one was found false-positive. Comparing with Ziehl-Neelsen stain, the sensitivity and specificity of PCR-Luminex assay were 90.7% (49/54) and 99.1% (108/109), respectively (Table 3). The results of PCR-Luminex assay and Ziehl-Neelsen stain for matching were compared by using kappa test (*k* = 0.9153). According to MGG stain, the “gold standard” for cryptococcal meningitis, 23 of 25 PCR-Luminex positive specimens were true positives and four were false positives. Our results demonstrated that the sensitivity and specificity for cryptococcal meningitis were 92% (23/25) and 97.1% (134/138), respectively (Table 4). The two methods showed good consistency (*k* = 0.8628), suggesting that PCR-Luminex assay matching was in agreement with MGG stain. Based on results from ELISA as the reference test, HSV-1 for PCR-Luminex assay had diagnostic sensitivity and specificity of 80.8% (21/26) and 94.2% (129/137), respectively (Table 5). The consistency check showed that k coefficient of the two methods was 0.7158. For HSV-2, one sample which was positive for ELISA was detected positive, and another sample for ELISA negative gave a positive result when tested with PCR-Luminex assay (Table 5). In addition, the *S. pneumoniae* subassay also identified five specimens with suspected CNS infections (Data not shown).

**Table 3:** *M. tuberculosis* detected by PCR-Luminex assay *vs.* indirect smear microscopy.

PCR-Luminex	Smear Positive	Smear Negative	Sensitivity	Specificity	Kappa
(+)	49	1	90.7%	99.1%	0.9153
(−)	5	108			

**Table 4:** *C.* *neoformans* detected by PCR-Luminex assay *vs.* MGG stain.

PCR-Luminex	MGG Positive	MGG Negative	Sensitivity	Specificity	Kappa
(+)	23	4	92%	97.1%	0.8628
(−)	2	134			

MGG, May Grunwald Giemsa stain.

**Table 5:** HSV-1 or HSV-2 detected by PCR-Luminex assay *vs.* ELISA assay.

PCR-Luminex		ELISA Positive	ELISA Negative	Sensitivity	Specificity	Kappa
HSV-1	(+)	21	8	80.8%	94.2%	0.7158
	(−)	5	129			
HSV-2	(+)	1	1	100%	99%	0.6639
	(−)	0	161			

ELISA, Enzyme linked immunosorbent assay.

## 4. Discussion

In this report, the Luminex-based strategy for multiplex detection of pathogen nucleic acids was a useful method for diagnosis of CNS infections, which could be utilized to identify five different pathogens in CSF specimens simultaneously. Using PCR-Luminex technique we examined the specificity and sensitivity of this assay, the results showed that this detection system had specificity and sensitivity comparable to the conventional diagnostic methods. Our present study also showed that the target PCR products were only hybridized with the oligonucleotide “anti-TAG” sequences on the corresponding microspheres in the testing system and non-specific reactions were not found, consistent with previous studies [25,35,36]. Moreover, we also found that the limit of detection sensitivity revealed detection of as low as 10^1^–10^2^ copies of plasmid DNAs. Hence, these findings suggest that the development of the multiplex detection platform based on PCR-Luminex technology for screening five pathogens is actionable. 

Previous studies [37,38,39,40] showed that the sensitivity and specificity of Luminex technology detection are mainly relied on the designing and synthesizing for specific primers and probes, which are the key and prerequisite for construction of gene chips. Specific primers can ensure the stability of the experiment and reduce non-specific reactions occurrences. Consequently, the GC content of the primers we designed in our experiments were all between 55% and 60%, and the annealing temperature were between 55 °C and 60 °C as well. Then, the results showed that all the primers were designed appropriately and ensured that the five target fragments could be amplified simultaneously. 

Wilson *et al*., [41] reported that ten-plex PCR-coupled liquid bead array detected lower sensitivity than the single PCR. Thus, it is indispensable for us to optimize the conditions of multiplex PCR reaction. To ensure the specificity and sensitivity of the assay, we also performed the optimizing experiments for hybridization reaction conditions and confirmed 37 °C was the optimal hybridization temperature and 30 minutes as the optimum hybridization time. Like with previous studies [23,26,42], using MagPlex-TAG microspheres in the experiment could guarantee the same annealing temperature, and effectively avoid cross-hybridization. A total of 163 CSF specimens collected over 10 years from patients with suspected CNS infections were examined, and we found 113 specimens were detected positive by PCR-Luminex analysis. Of these, 94 (88.7%) could be defined as true-positive, which were positive by conventional diagnostic methods as well as the PCR-Luminex assay. When the performance of this assay was assessed with clinical specimens previously tested positive by conventional methods, we found that the assay had a relatively high diagnostic accuracy for tuberculosis meningitis, cryptococcal meningitis and HSV-1 and HSV-2 encephalitis, respectively. However, the CSF specimens had been stored at −20 °C for a long time (years) and a consequent false negative PCR-Luminex assay result might offer a likely explanation. Furthermore, the cutoff values were instead set by a practical method based on the values for the NTCs and based on the positive results for the samples by the conventional assay [33]. This might also lead to impaired sensitivity. 

The multiplex PCR-Luminex system could simultaneously detect five pathogen nucleic acids in a single reaction and provide a high-throughput low-time consuming assay. Admittedly, in the present study the use of only only one primer set for each target may raise the possibility of false-negative results [35]. Like conventional PCR with open stages, the limitation of this approach is the potential to produce false-positive results due to cross-contamination with amplicons from the environment. Another drawback of the evaluation of the method was that we used frozen CSF specimens that had been stored for a long period of time, which may lead to an impaired sensitivity. The sensitivity would presumably be improved if fresh CSF specimens were used. Considering these, multiply primer sets, implementing standardized PCR laboratory procedures, increasing sample sizes, and specimen collection handling and processing should be adopted or improved in our future study.

## 5. Conclusions 

Here we established a rapid and sensitive five-plex PCR-Luminex assay for simultaneous detection of five pathogens from CSF specimens in 3.5 h. This approach might be a promising supplement to the conventional methods for diagnosing CNS infections.
